# Supplementary material for: Antigen recognition reinforces regulatory T cell mediated Leishmania major persistence
Source: Nat Commun. 2023 Dec 19;14:8449. doi: 10.1038/s41467-023-44297-6 (PMC10730873; doi:10.1038/s41467-023-44297-6)
Supplement: Supplementary file 1 — Supplementary Information [file 41467_2023_44297_MOESM1_ESM.pdf]

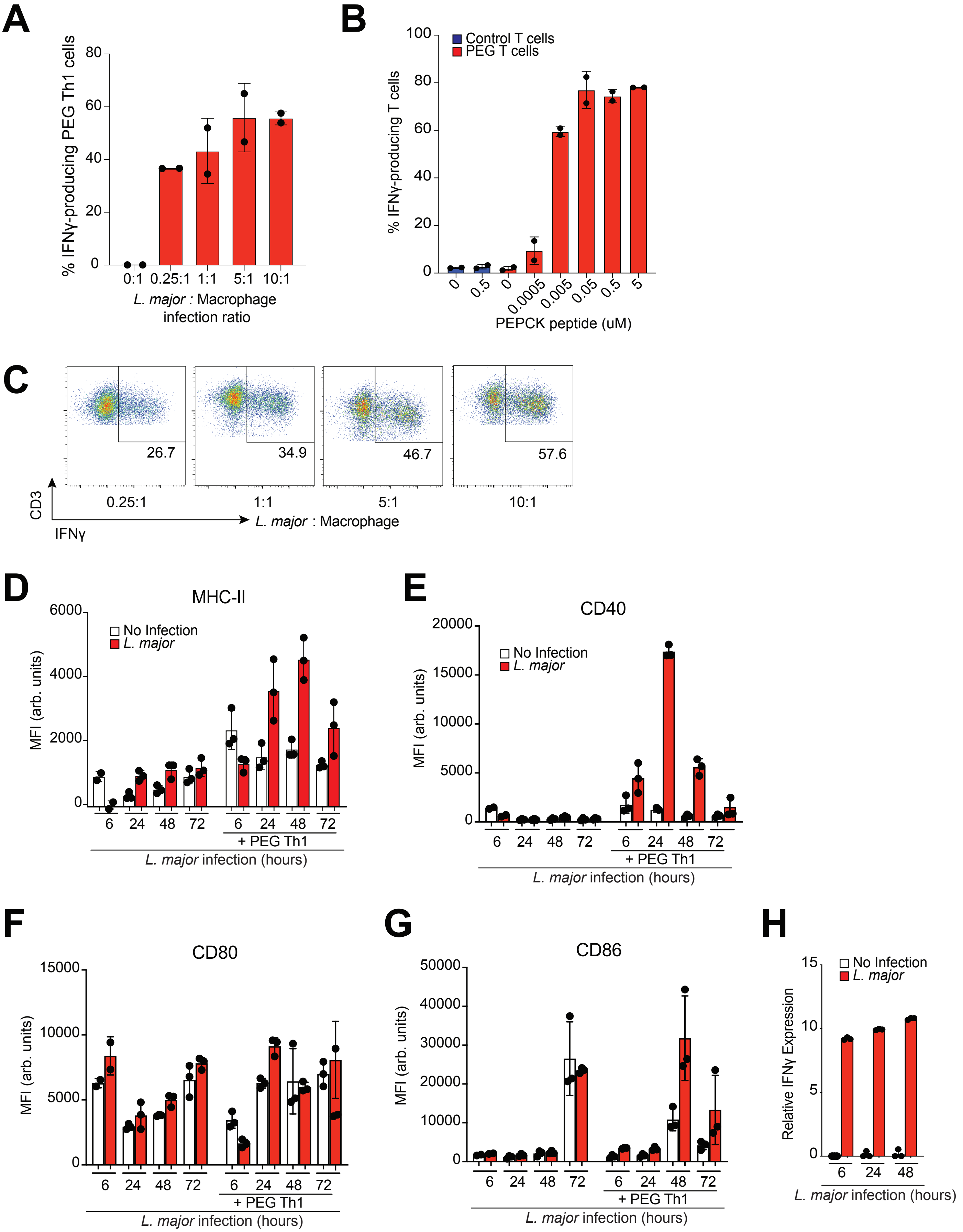

Supplementary Figure 1. IFNγ dose response and time-course phenotyping of *L. major*-infected macrophages
(A) IFNγ production after varying ratios of L.major infected macrophages. (B) Control or PEG Th1 cells were co-cultured with PEPCK335-351-pulsed macrophages and IFNγ production by Th1 cells was measured by Flow cytometry. Statistical analysis– unpaired t test. Mean +/- SD. Representative data of three independent experiments. (C) PEG Th1 cells were co-cultured with *L. major*-infected macrophages infected at various parasite: macrophage ratios and IFNγ production by Th1 cells was measured by Flow cytometry. (D-G) Cell surface MHC-II, CD40, CD80, and CD86 expression on macrophages was measured with flow cytometry over time with (red) or without (white) *L. major* infection in the absence or presence of PEPCK Th1 cells. Mean +/- SD. Representative data of two independent experiments. (H) Relative production by PEG Th1 cells was measured by flow cytometry upon co-culture with *L. major*-infected macrophages at 6,24, and 48 hours post infection. Mean +/- SD. Data representative of two independent experiments.

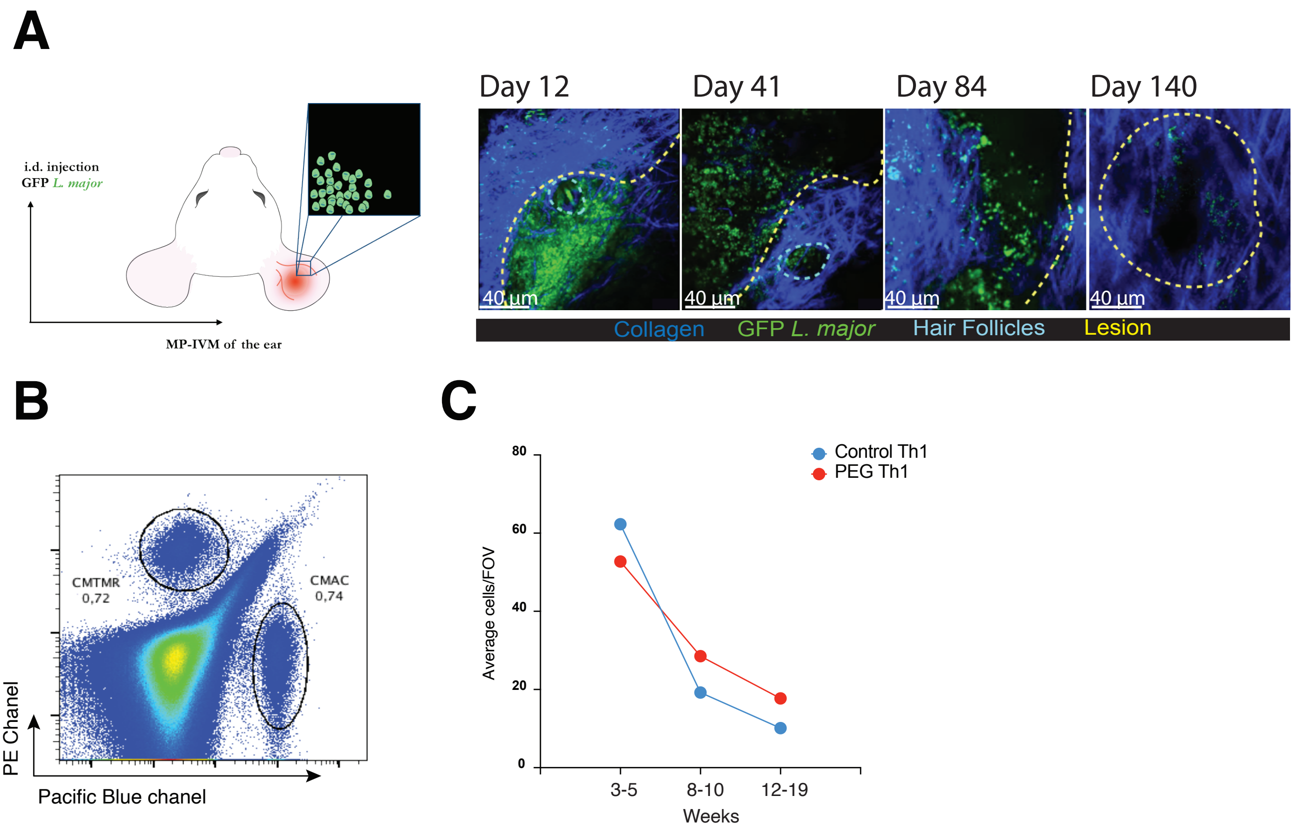

Supplementary Figure 2. ***Leishmania major*** **parasites persist in the ear pinna**.
(A) Albino C57BL/6 mice were infected intradermally in the ear pinna with 1 million GFP *L. major* parasites and intravital microscopy was performed on ear dermis at various time points. Collagen – blue, GFP L major – green, yellow dotted line represents lesion area, blue dotted line represents a hair follicle. (B) Flow Cytometry analysis of CMAC (cell tracker blue) and CMTMR (cell tracker red) stained Th1 cells in the spleen posts adoptive transfer and intravital microscopy studies. (C) Average number of control (blue) and PEG (red) cells per recording. FOV:field of view.

Supplementary Figure 2.

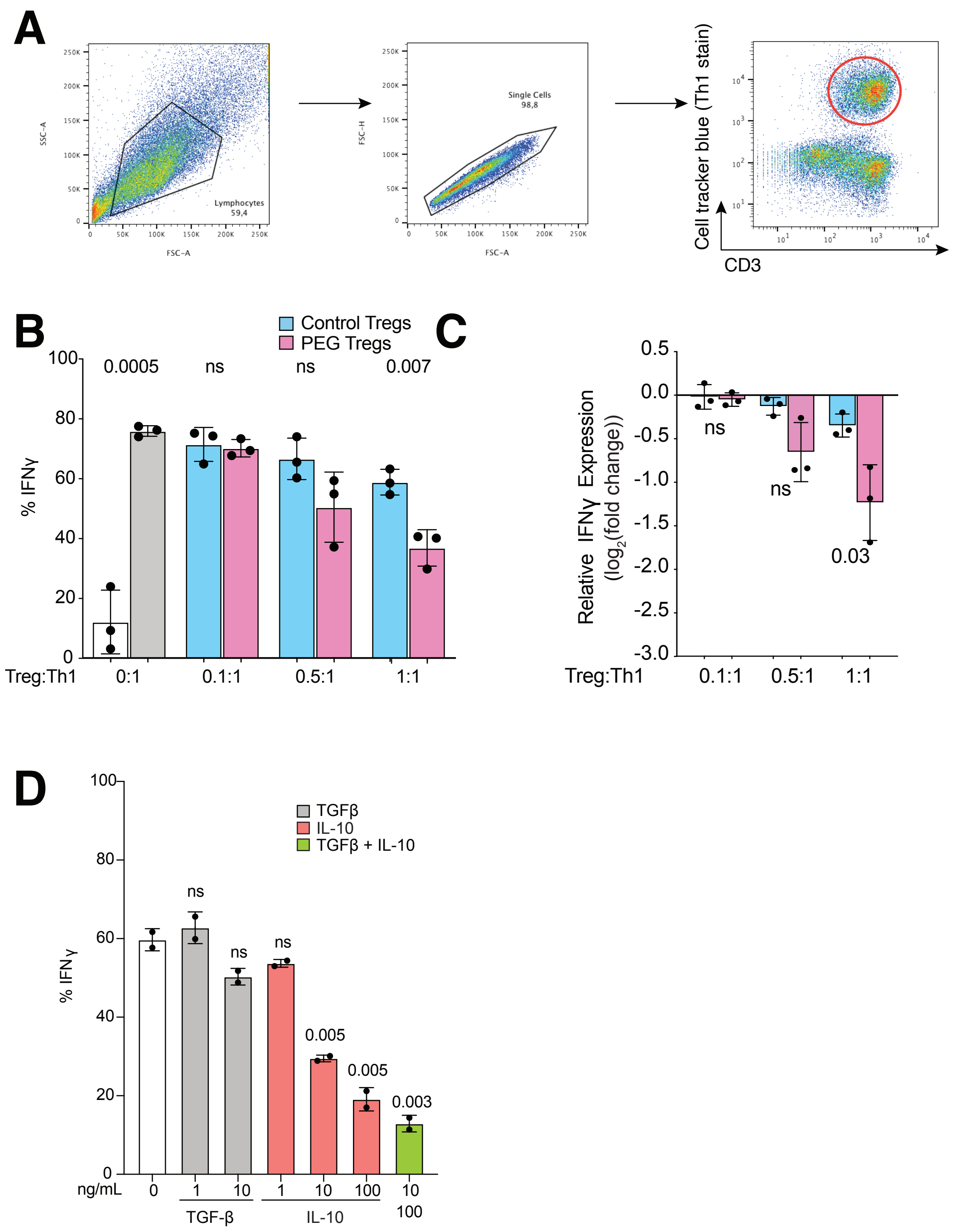

Supplementary Figure 3. Treg-driven suppression of IFNγ production by PEG Th1 cells
(A) Gating strategy for selecting cell tracker blue-stained PEG Th1 cells from the co-culture with *L. major*-infected macrophages and either Control or PEG Tregs. (B) IFNγ production by PEG Th1 cells was measured with flow cytometry at various Th1:Treg ratios in response to macrophages pulsed with 0.5 μM PEPCK335-351 peptide. Each dot represents a mean of 3 experimental replicates. Mean +/- SEM, Statistical analysis – unpaired t test, ns = not significant. Data combined from three independent experiments.(C) Relative IFNγ production by PEG Th1 cells at various Th1:Treg ratios in response to macrophages pulsed with 0.5 μM PEPCK335-351 peptide compared to 0:1 Treg: Th1 ratio. Each dot represents a mean of 3 experimental replicates. Two-tailed unpaired student's *t*-test, ns = not significant. Mean +/- SEM. Data combined from three independent experiments. (D) IFNγ production by PEG Th1 cells was measured by flow cytometry in the indicated conditions. Representative data from two independent experiments, each dot represents an experimental replicate. Mean+/- SD. ns = not significant.

Supplementary Figure 3.

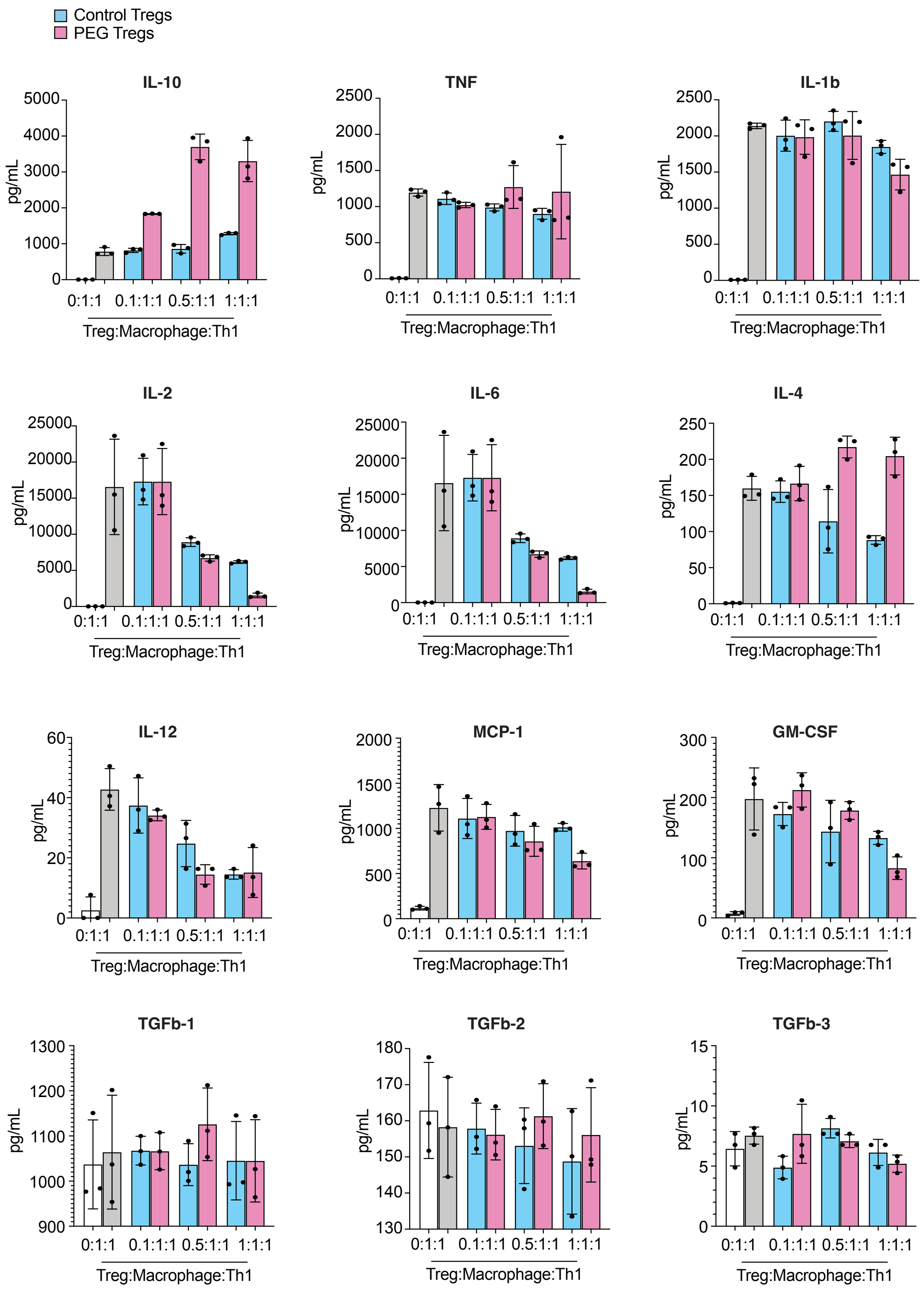

Supplementary Figure 4. ELISA measurements of the pro-inflammatory cytokine panel. Measurements of supernatants from co-cultures of PEPCK Th1 cells and *L. major*-infected macrophages (Gray bar), and with the addition either control (blue) or PEPCK (pink) Tregs. Each dot represents a single experimental replicate. Representative data of two independent experiments.
