# Supplementary material for: Antigen recognition reinforces regulatory T cell mediated Leishmania major persistence
Source: Nat Commun. 2023 Dec 19;14:8449. doi: 10.1038/s41467-023-44297-6 (PMC10730873; doi:10.1038/s41467-023-44297-6)
Supplement: Supplementary file 3 — Description of Additional Supplementary Files [file 41467_2023_44297_MOESM3_ESM.pdf]

**Description of Additional Supplementary Files**

File Name: Supplementary Movie 1
Description: **Control and PEG Th1 cells behaviour in *Leishmania major*-infected skin, related to Figure 2.** CMTRM-labeled control Th1 cells (red) and CMAC-labeled PEG Th1 cells (blue) were adoptively transferred into *GFP L. major* (green) -infected C57BL/6 mice at either week 3 or Week 15 post infection. Intravital microscopy was performed 1 day after transfer. Each individual frame is a maximum intensity projection of 12 z stacks spaced 4 µm apart (total thickness of 44 µm). Scale bar = 50 µm. Time is shown in min:sec elapsed of the movie recording.

File Name: Supplementary Movie 2
Description: **PEG Th1 cells form prolonged contacts with *Leishmania major*-infected BMM, related to Figure 4.** CMFDA-labeled control Th1 cells (green) and CMTMR-labeled PEG Th1 cells (red) together with uninfected or *GFP L. major* (green) -infected CMAC-stained BMM (blue) were embedded into collagen gels for live-cell imaging. Each individual frame is a maximum intensity projection of 12 z stacks spaced 4 µm apart (total thickness of 44 µm). Scale bar = 50 µm. Time is shown in min:sec elapsed of the movie recording.

File Name: Supplementary Movie 3
Description: **Tregs do not disrupt PEG Th1: *L. major*-infected BMM contacts.** CMTMR-labeled PEG Th1 cells (red) and either CMFDA-labeled control or PEG Tregs (green) together with *L. major* -infected CMAC-stained BMM (blue) were embedded into collagen gels for live-cell imaging. Each individual frame is a maximum intensity projection of 12 z stacks spaced 4 µm apart (total thickness of 44 µm). Scale bar = 50 µm. Time is shown in min:sec elapsed of the movie recording.

File Name: Supplementary Movie 4
Description: ***In vivo* expansion of *L. major*-specific Tregs leads to disease reactivation, related to Figure 5.** Foxp3-GFP (green) C57BL/6 mice were infected with dsRed *L. major* (red) in the ear pinna and allowed to heal for 60 days. Healed mice were then challenged with heat-killed wild-type or PEPCK$^{-/-}$ *L. major* by footpad injection, and intravital microscopy performed at day 14. Each individual frame is a maximum intensity projection of 12 z stacks spaced 4 µm apart (total thickness of 44 µm). Scale bar = 100 µm. Time is shown in min:sec elapsed of the movie recording.
