# Supplementary material for: Antigen recognition reinforces regulatory T cell mediated Leishmania major persistence
Source: Nat Commun. 2023 Dec 19;14:8449. doi: 10.1038/s41467-023-44297-6 (PMC10730873; doi:10.1038/s41467-023-44297-6)
Supplement: Supplementary file 8 — Reporting Summary [file 41467_2023_44297_MOESM8_ESM.pdf]

nature portfolio

Corresponding author(s): Thomas Murooka, Jude Uzonna

Last updated by author(s): Nov 20, 2023

Reporting Summary

Nature Portfolio wishes to improve the reproducibility of the work that we publish. This form provides structure for consistency and transparency in reporting. For further information on Nature Portfolio policies, see our Editorial Policies and the Editorial Policy Checklist.

Statistics

For all statistical analyses, confirm that the following items are present in the figure legend, table legend, main text, or Methods section.

n/a	Confirmed	
☐	☒	The exact sample size (*n*) for each experimental group/condition, given as a discrete number and unit of measurement
☐	☒	A statement on whether measurements were taken from distinct samples or whether the same sample was measured repeatedly
☐	☒	The statistical test(s) used AND whether they are one- or two-sided<br>*Only common tests should be described solely by name; describe more complex techniques in the Methods section.*
☒	☐	A description of all covariates tested
☒	☐	A description of any assumptions or corrections, such as tests of normality and adjustment for multiple comparisons
☐	☒	A full description of the statistical parameters including central tendency (e.g. means) or other basic estimates (e.g. regression coefficient) AND variation (e.g. standard deviation) or associated estimates of uncertainty (e.g. confidence intervals)
☐	☒	For null hypothesis testing, the test statistic (e.g. F, t, r) with confidence intervals, effect sizes, degrees of freedom and P value noted<br>*Give P values as exact values whenever suitable.*
☒	☐	For Bayesian analysis, information on the choice of priors and Markov chain Monte Carlo settings
☒	☐	For hierarchical and complex designs, identification of the appropriate level for tests and full reporting of outcomes
☒	☐	Estimates of effect sizes (e.g. Cohen's *d*, Pearson's *r*), indicating how they were calculated

Our web collection on statistics for biologists contains articles on many of the points above.

Software and code

Policy information about availability of computer code

Data collection	No software was used
Data analysis	Bitplane Imaris 8.0 was used to measure 3D migration speeds and confinement ratios. FlowJo was used to analyze all flow cytometry data.

For manuscripts utilizing custom algorithms or software that are central to the research but not yet described in published literature, software must be made available to editors and reviewers. We strongly encourage code deposition in a community repository (e.g. GitHub). See the Nature Portfolio guidelines for submitting code & software for further information.

Data

Policy information about availability of data

All manuscripts must include a data availability statement. This statement should provide the following information, where applicable:

- Accession codes, unique identifiers, or web links for publicly available datasets
- A description of any restrictions on data availability
- For clinical datasets or third party data, please ensure that the statement adheres to our policy

The source data for each graph (Main and supplementary figures) are provided as a Source data file. The source data for each graph (Main and supplementary figures) are provided as a Source data file. Any additional inquiries should be addressed to Thomas T. Murooka or Jude E. Uzonna (corresponding authors).

Research involving human participants, their data, or biological material

Policy information about studies with human participants or human data. See also policy information about sex, gender (identity/presentation), and sexual orientation and race, ethnicity and racism.

Reporting on sex and gender	n/a
Reporting on race, ethnicity, or other socially relevant groupings	n/a
Population characteristics	n/a
Recruitment	n/a
Ethics oversight	n/a

Note that full information on the approval of the study protocol must also be provided in the manuscript.

Field-specific reporting

Please select the one below that is the best fit for your research. If you are not sure, read the appropriate sections before making your selection.

☒ Life sciences ☐ Behavioural & social sciences ☐ Ecological, evolutionary & environmental sciences

For a reference copy of the document with all sections, see nature.com/documents/nr-reporting-summary-flat.pdf

Life sciences study design

All studies must disclose on these points even when the disclosure is negative.

Sample size	No predetermined sample size calculations were performed. In most experiment, 3-5 mice were used for each condition, with 2-3 repeat experiments performed and used the perform statistical analyses between relevant study groups (which is indicated in the figure legends).
Data exclusions	no data was excluded from the analysis
Replication	all studies were repeated 2-3 independent times. In some cases, data from all 3 experiments are shown, whereas in others a representative graph from one study is displayed. This is clearly stated in the figure legends.
Randomization	All animals imported into the facility was randomly assigned to different cages by the animal technician(s) who are not involved in the experimental studies.
Blinding	This work was primarily led by the lead author and blinding was not possible in all experiments. In many cases, parasite infection and associated clinical parameters of disease can be determined by the researcher even under blinding. Efforts were made to keep the tissue analysis blinded by assigning code names to samples by a research technician during microscopy.

Reporting for specific materials, systems and methods

We require information from authors about some types of materials, experimental systems and methods used in many studies. Here, indicate whether each material, system or method listed is relevant to your study. If you are not sure if a list item applies to your research, read the appropriate section before selecting a response.

Materials & experimental systems

n/a	Involved in the study	
☐	☒	Antibodies
☒	☐	Eukaryotic cell lines
☒	☐	Palaeontology and archaeology
☐	☒	Animals and other organisms
☒	☐	Clinical data
☒	☐	Dual use research of concern
☒	☐	Plants

Methods

n/a	Involved in the study	
☒	☐	ChIP-seq
☐	☒	Flow cytometry
☒	☐	MRI-based neuroimaging

Antibodies

Antibodies used	The cells were directly stained ex vivo for surface expression of CD4 (clone GK1.5, Biolegend cat #100401), CD45 (biolegend clone S18009D cat#160302), CD3 (clone 17A2 cat #100201), CD25 (clone PC61 cat #102002), foxp3 (clone 3G3; eBioscience Cat # 00-5523-00) and T-bet (clone 4B10; biolegend cat #644801).<br><br>For IHC, the following was used: The primary antibody used was rat anti-F4/80 (Abcam cat#ab6640) at 1:500 dilution. Secondary antibodies used were AF568-conjugated goat anti-rat (Abcam cat#ab175476) at 1:1000 dilution, and AF488 conjugated chicken anti-GFP (Abcam cat#ab13970) at 1:5000 dilution.
Validation	All antibodies used were from commercial sources and validated by the company.<br>Biolegend (www.biolegend.com) for validation information on all flow cytometry antibody clones purchased from this vendor.<br>EBioscience (www.thermofisher.com) for validation information on the anti-foxp3 antibody and flow cytometry staining kits.<br>Abcam (www.abcam.com) for validation information of antibodies used for IHC.

Animals and other research organisms

Policy information about studies involving animals; ARRIVE guidelines recommended for reporting animal research, and Sex and Gender in Research

Laboratory animals	wildtype C57Bl/6J mice, males ages 6-8 weeks old purchased from the University of Manitoba internal breeding colony or Jackson Labs (stock 000664)<br><br>albino C57Bl/6J (Cg) Tyr c-2J/J mice, males ages 6-8 weeks old purchased from Jackson Labs (stock 000058)<br><br>Foxp3-GFP mice (B6Cg-Foxp3tm2Tch Homozygous), males ages 6-8 weeks old purchased from Jackson Labs (stock 006772)<br><br>PEPCK TCR-transgenic mice on the C57LB/6J background were generated and acquired from an in-house breeding colony at the University of Manitoba.
Wild animals	no wild animals were used in this study.
Reporting on sex	All experiments were performed using mouse or mouse-derived cells, which were all males in this study. This was to eliminate the variability induced by fluctuations in sex hormones, but remains a limitation of this study in terms of broad applicability of T cell responses to both sexes. Future studies will compare T cell responses reported here between male and female recipients.
Field-collected samples	No field collected samples were used in this study.
Ethics oversight	Animal approval through the University of Manitoba animal care committee in accordance with the Canadian Council for Animal Care guidelines, protocol #21-022.

Note that full information on the approval of the study protocol must also be provided in the manuscript.

Plants

Seed stocks	n/a
Novel plant genotypes	n/a
Authentication	n/a

Flow Cytometry

Plots

Confirm that:

☒ The axis labels state the marker and fluorochrome used (e.g. CD4-FITC).

☒ The axis scales are clearly visible. Include numbers along axes only for bottom left plot of group (a 'group' is an analysis of identical markers).

☒ All plots are contour plots with outliers or pseudocolor plots.

☒ A numerical value for number of cells or percentage (with statistics) is provided.

Methodology

Sample preparation	Cells from mouse tissues such as ears, spleen and bone marrow was prepared into a single-cell suspension and stained using the indicated antibodies according to manufacturer's protocols. Cells were then prepared for flow cyometry on the same day as processing and staining.
Instrument	BD FACS Canto-II
Software	FACS DIVA, FlowJo
Cell population abundance	no sorting was performed in this study
Gating strategy	Provided in main or supplementary figure, where appropriate

☒ Tick this box to confirm that a figure exemplifying the gating strategy is provided in the Supplementary Information.
